# Endotracheal intubation-related oral mucosal membrane pressure injuries: a narrative review of biomechanical insights, biomaterial optimization, and intelligent monitoring

**DOI:** 10.3389/fmedt.2025.1667748

**Published:** 2025-11-21

**Authors:** Limei Cai, Yijing Li, Yonggang Liu, Guo Ma, Qinfang Zhang, Xiaoxi Li, Na Li

**Affiliations:** 1 Department of Intensive Care Medicine, The First Affiliated Hospital of Kunming Medical University, Kunming, Yunnan, China; 2 Department of Dermatology, The First Affiliated Hospital of Kunming Medical University, Kunming, Yunnan, China

**Keywords:** orotracheal intubation, oral mucosal membrane pressure injury, microecosystem regulation, orotracheal, oral mucosal membrane

## Abstract

**Objectives:**

This article is a narrative review that synthesizes current evidence on orotracheal intubation-related oral mucosal membrane pressure injuries in intensive care unit (ICU) patients, focusing on mechanisms, risk factors, and prevention strategies. The review is intended to inform clinicians and researchers by integrating insights from intensive care, biomechanics, biomaterials, and oral microbiology.

**Methods:**

A comprehensive literature search was conducted in PubMed, Web of Science, Embase, and CNKI using the terms “orotracheal intubation”, “oral mucosal injury”, “device-related pressure injury”, “biomechanics”, “biomaterials” and “oral microbiome”. Studies published between 2000 and 2025, including both clinical and experimental research, were considered without language restrictions.

**Results:**

Evidence indicates that vertical pressure, shear force, and friction from endotracheal tubes are key contributors to oral mucosal injury. Reported risk factors include advanced age, prolonged intubation, malnutrition, and inflammation. Preventive strategies have been explored in four domains: biomechanical modeling using finite element analysis, biomaterial optimization such as hydrogel and nanocoatings, regulation of the oral microecosystem through probiotics, and intelligent monitoring systems incorporating artificial intelligence and Internet of Things technologies.

**Conclusions:**

Orotracheal intubation-related oral mucosal pressure injuries are multifactorial and preventable. This narrative review integrates biomechanical insights, optimized biomaterials, microbiome regulation, and intelligent monitoring into a multidimensional prevention framework. Such strategies may enhance early identification, reduce complications, and improve clinical outcomes in ICU patients.

## 1 Introduction

In recent years, critically ill patients admitted to the intensive care unit (ICU) have presented with complex and rapidly changing conditions, often requiring various invasive procedures, such as artificial airway placement for mechanical ventilation (1). Advancements in critical care and airway management have established orotracheal intubation with mechanical ventilation as a routine respiratory support strategy for critically ill patients in the ICU. Previous multicenter investigations have shown that approximately 39% of ICU patients required mechanical ventilation, with subsequent studies reporting proportions ranging from 39% to 70% (2, 3). Artificial airway placement for mechanical ventilation has been recognized as a key therapeutic approach in the management of critically ill patients in the ICU. The artificial airway serves not only as the primary pathway for respiratory support in critically ill patients but also as a key and effective intervention during resuscitation, ensuring both airway patency and patient survival (4).

Artificial airways can be established through three methods, including nasotracheal intubation, orotracheal intubation, and tracheotomy. Among these, orotracheal intubation is the most frequently used in clinical settings (5), representing over 96% of all tracheal intubations (6) Compared with the other methods, orotracheal intubation is easier to master and more convenient to perform (7). Despite its effectiveness in improving respiratory function in critically ill patients, orotracheal intubation with mechanical ventilation introduces additional challenges to nursing management.

In addition, the concept of device-related pressure injuries (DRPI) has been proposed in the literature (8), referring to localized injuries of the skin, subcutaneous tissues, or mucosa resulting from external medical device pressure. These injuries usually exhibit a shape similar to that of the responsible device. A survey conducted by Hanonu (9) indicated that DRPI accounted for approximately 30% of all pressure injuries, and an international investigation in intensive care unit (ICU) settings reported that DRPI constituted 43.5% of hospital-acquired pressure injuries (10). DRPI includes both conventional skin pressure injuries and mucosal membrane pressure injuries (MMPI). Some research institutions have defined MMPI as localized mucosal damage caused by medical devices (11). Unlike traditional skin pressure injury sites, internal mucosa compressed by invasive catheters is more prone to MMPI (12). Studies have shown that among MMPI caused by invasive catheters, oral MMPI associated with orotracheal intubation are the most common (13). ICU patients with critical conditions frequently undergo prolonged orotracheal intubation, during which oral mucosal examination is challenging, as it requires removal of the bite block and opening of the mouth. As a result, MMPI frequently develop at the contact sites between the orotracheal tube or its fixation devices and the oral mucosa. These injuries are typically located in concealed areas and are often not recognized until bleeding, ulceration, or mucosal breakdown occurs (13–15). A survey conducted by Amrani (16) reported that the incidence of orotracheal intubation-related oral MMPI in ICU patients reached as high as 45%, significantly higher than that in general wards. The emergency ICU (EICU) is part of the critical care system while also exhibiting the characteristics of emergency medicine. Due to the complex spectrum of diseases, an increasing proportion of elderly patients (17), insufficient staffing, and heavy workloads, the incidence of orotracheal intubation-related oral MMPI in EICU patients is higher than that in general wards (18). The study by Ning et al. (19) revealed that oral MMPI occurred in 30.16% of EICU patients who received orotracheal intubation.

Oral MMPI caused by orotracheal intubation may result in pain, infection, tissue adhesion, functional disorders, financial burden, and increased workload for medical staff. These consequences reduce patient and family satisfaction, impair nurse-patient relationships, and may trigger occupational issues and adverse events, ultimately increasing patient distress, negatively affecting prognosis, extending hospitalization, and increasing complaints against healthcare personnel and institutions (16–18, 20). According to investigations, traditional intubation care strategies primarily rely on improvements in fixation methods, topical lubricants, or routine oral care. However, these strategies are insufficient for comprehensive prevention and effective healing.

Given the high incidence and clinical impact of tracheal intubation-related oral mucosal injuries, a comprehensive understanding of their biomechanical mechanisms, risk factors, and preventive strategies is essential. This review synthesizes current research on injury prediction using finite element analysis (FEA), biomaterial optimization through hydrogel and nanocoatings, and oral microecological regulation. Furthermore, it explores intelligent monitoring systems and personalized care strategies to enhance early prevention and clinical management. The scope of this review is therefore limited to these four domains, which together provide a multidimensional framework for understanding and preventing oral mucosal pressure injuries associated with orotracheal intubation. This manuscript is intended for clinicians, nurses, and researchers in intensive care, dentistry, and biomaterials science, aiming to provide an interdisciplinary synthesis of current knowledge and practical strategies.

## 2 Mechanisms of oral mucosal membrane pressure injuries associated with tracheal intubation

### 2.1 Tissue injury caused by mechanical stress

**Figure 1:**
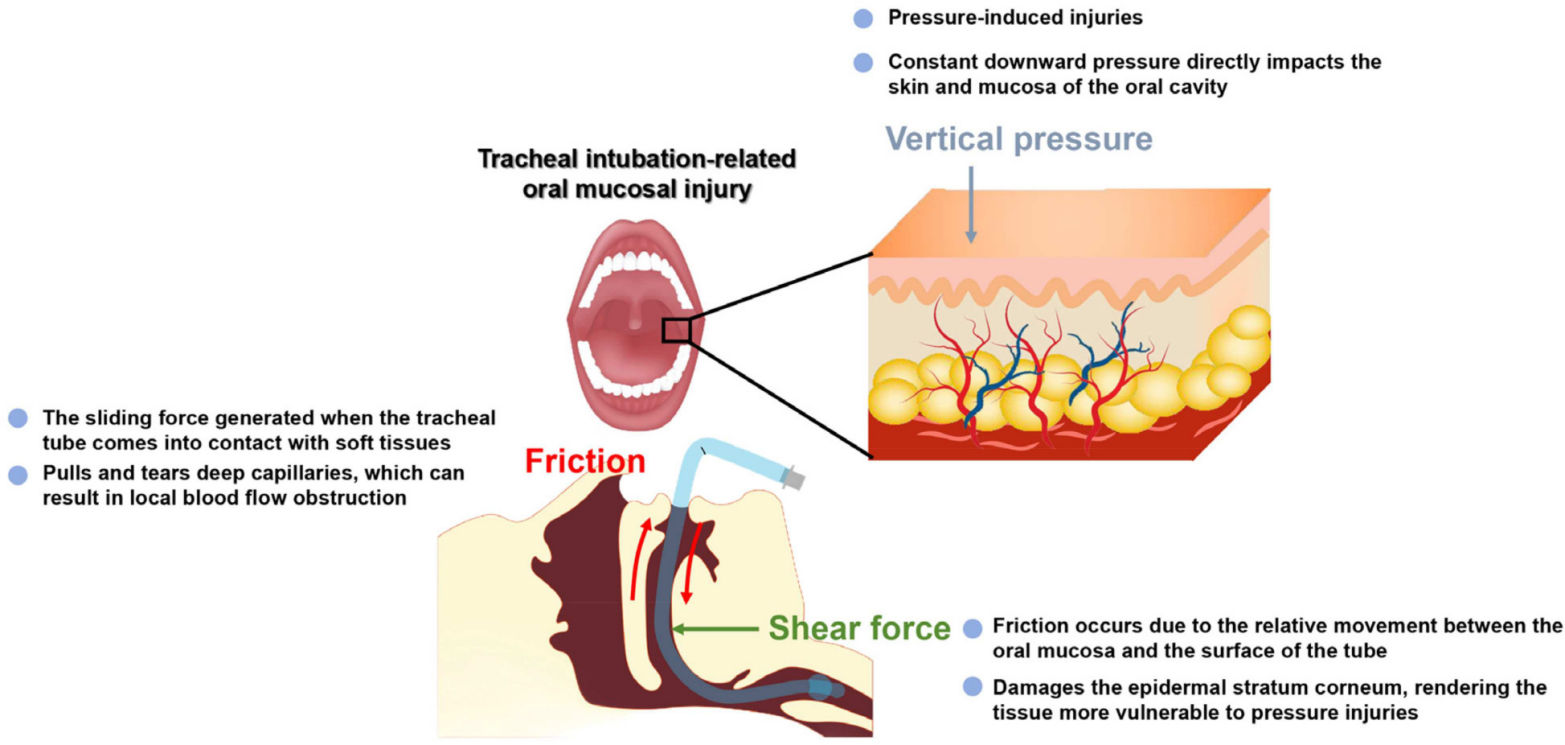
Mechanisms of tracheal intubation-related oral mucosal injury. The diagram illustrates the three primary mechanical forces contributing to mucosal damage: vertical pressure, friction, and shear force. This schematic is illustrative and does not represent the exact anatomical structure of the oral mucosa.

Endotracheal intubation is essential for mechanical ventilation in ICU patients; however, both the endotracheal tube and improper fixation methods may contribute to the development of oral MMPI (21). From a biomechanical perspective, oral MMPI caused by endotracheal tubes are primarily attributed to vertical pressure, shear force, and friction (22) (Figure 1). Sustained vertical pressure directly applied to the skin and mucosal tissues is the main cause of pressure injuries. The occurrence of oral MMPI is closely related to both the intensity and duration of pressure, with higher pressure levels and longer exposure times increasing the likelihood of oral MMPI (23). In addition, inappropriate tube size and excessive fixation tension can further increase both pressure and shear forces (24). Research has indicated that shear force refers to the force applied to the surfaces of adjacent tissues, causing them to slide in opposite directions. Shear force is generated by the combination of pressure and friction, and occurs when the endotracheal tube contacts the soft tissues of the oral cavity. When shear force acts on the deeper layers of the skin, it can stretch, distort, and tear capillaries, interrupting local blood flow, leading to impaired circulation and deep tissue necrosis. Such injuries are difficult to be detected in the early stages, and tissue damage caused by shear force is more challenging to heal than typical wounds (22). Friction arises from the relative movement between the oral mucosa and the endotracheal tube surfaces. Although friction does not directly cause oral MMPI, it can damage the stratum corneum of the oral mucosa, leading to superficial mucosal exfoliation and increased susceptibility to pressure injuries. Once the damaged oral mucosa is exposed to saliva and other secretions, the risk of pressure injury is further exacerbated. Moreover, friction increases the local temperature of the mucosal tissues, disrupting the microenvironmental balance, altering pH levels, and increasing oxygen consumption, which further exacerbates tissue ischemia and elevates the risk of oral MMPI (23).

### 2.2 Influencing factors of oral mucosal membrane pressure injuries associated with endotracheal intubation

The occurrence of oral MMPI in ICU patients with endotracheal intubation is influenced by multiple interrelated factors,including patient factors, physiological factors, biomechanical factors, the use of specific medications, and nursing-related factors (Table 1) (25). Patient-related factors such as advanced age, severe illness, impaired consciousness, reduced physical activity, and malnutrition increase the risk of MMPI by compromising tissue perfusion and sensory perception, which reduces the ability to detect and respond to sustained pressure (26). Additionally, disruptions in the local microenvironment, including changes in oral microbiota and moisture levels, further predispose the mucosa to injury (27).

**Table 1:** Influencing factors of orotracheal intubation-related oral mucosal membrane pressure injuries.

Classification	Detailed factors	Influencing mechanism
Patient factors	Age	In elderly individuals, the oral mucosa is atrophic and less elastic, making it more susceptible to injury.
Disease-related factors	Low hemoglobin/serum albumin concentrations	Patients with low serum albumin levels have a higher risk of injury.
Treatment-related factors	Prone ventilation; critically ill condition; extracorporeal membrane oxygenation	Prone ventilation and the use of vasoactive agents may increase the risk of injury by impairing local blood circulation or increasing localized pressure; Critical illness and hemodynamic instability further contribute to the risk of injury; Additionally, inflammatory responses during ECMO therapy increase microvascular permeability, leading to tissue and mucosal edema.
Friction and shear force	Pressure injury caused by internal tissue response to external mechanical loading on soft tissue	The size of the endotracheal tube, duration of intubation, and technical proficiency directly affect mucosal pressure and the risk of injury.

ECMO, extracorporeal membrane oxygenation.

Physiological factors, including white blood cell count, C-reactive protein, interleukin-6, serum albumin, hemoglobin, and hematocrit levels, play a crucial role in tissue integrity and wound healing, with both excessive inflammation and impaired immune responses contributing to MMPI development. Biomechanical factors such as the rigidity of the medical device, improper fixation techniques, and prolonged intubation duration can exacerbate tissue damage. Inappropriate tube sizing—whether too large, causing excessive pressure, or too small, leading to tube displacement and friction—further increases injury risk (25).

The use of specific medications, including sedatives, analgesics, vasoactive agents, and corticosteroids, may also contribute to MMPI by reducing tissue sensitivity, lowering the pain threshold, and impairing local circulation through vasoconstriction, thereby promoting ischemia and delayed healing (16). Furthermore, nursing-related factors, including nurses' understanding, assessment, and prevention of oral MMPI, as well as the frequency of oral care and the application of relevant solutions, are associated with the development of oral MMPI. Multiple factors contribute to the development of oral MMPI in ICU patients (28). Given the multifactorial nature of MMPI, further prospective studies are warranted to better understand these risk factors and develop targeted prevention strategies.

### 2.3 Patient factors

Elderly patients are at high risk for DRPI (29). In elderly individuals, the skin and mucous membranes become thinner and less elastic as a result of water and nutrient loss. In addition, the presence of multiple comorbidities, reduced immune function, and delayed wound healing further increase the susceptibility of older patients to DRPI (29). Previous research has confirmed a positive correlation between older age and the risk of labial pressure injuries in ICU patients (30). In contrast, other studies have revealed no statistically significant difference in the incidence of orotracheal intubation-related oral MMPI across different age groups (31). Variations in study design, including sample size and patient selection, may account for these inconsistent findings.

## 3 Disease-related factors

### 3.1 Low hemoglobin concentration

Studies have shown that the risk of pressure injuries in patients with anemia is 2.23 times higher than in those with normal hemoglobin levels (32). However, opposing findings have been reported in studies on intubation-related oral MMPI, where hemoglobin concentration was positively correlated with the occurrence of upper oral MMPI (33). Therefore, the exact impact of hemoglobin levels requires further investigation.

### 3.2 Low serum albumin concentration

Serum albumin is the primary component of total serum protein and plays a vital role in maintaining blood colloid osmotic pressure, metabolite transport, and nutritional balance. Studies have identified low serum albumin levels as an independent risk factor for pressure injuries in ICU patients, which can also worsen pressure injuries and delay wound healing (34). However, other studies have reported no association between low serum albumin concentration and oral MMPI (33). Therefore, the relationship between these two variables requires further investigation.

## 4 Treatment-related factors

### 4.1 Prone ventilation

During prone ventilation, the oral mucosa is subjected to dual compression from the external support surface and the internal endotracheal tube or its fixation system. To date, no studies have investigated prone ventilation as a potential risk factor for oral MMPI. However, research on nasal mucosal pressure injuries has identified prone ventilation as an independent risk factor for nasal mucosal pressure injuries in ICU patients (35).

### 4.2 Surgery

In ICU patients, severe illness, hemodynamic instability, and substantial intraoperative blood loss may result in insufficient tissue reperfusion, increasing the risk of DRPI. Studies by Wu Dan (29) and Hu Jiehong et al. (36) reported similar findings, indicating a positive correlation between intraoperative blood loss and pressure injury scores. Gao et al. (16) also identified prolonged surgical duration (26 h) as an independent risk factor for pressure injury.

### 4.3 Extracorporeal membrane oxygenation

Extracorporeal membrane oxygenation (ECMO) is mainly applied in the management of cardiopulmonary insufficiency (37), providing additional time for the diagnosis and treatment of the primary disease (38). Currently, no studies have examined the relationship between ECMO and orotracheal intubation-related oral MMPI. However, considering that the inflammatory response during ECMO increases microvascular permeability, leading to mucosal edema (39), and based on clinical experience, ECMO was included as a potential influencing factor in this study.

## 5 Biomechanical, molecular, and pathophysiological mechanisms of oral mucosal epithelial barrier disruption

Studies have shown that pressure injuries develop due to the internal response of soft tissues to external mechanical loads. Mechanical loads include all types of forces applied to soft tissues due to contact between the skin and medical devices (40), with gravitational force transmitted through the endotracheal tube being the primary load exerted on the soft tissue. External mechanical loads are typically described as pressure (forces perpendicular to the skin surface) or shear forces (forces parallel to the skin surface). In clinical practice, the forces involved are a combination of pressure and shear force (41). Studies have shown that normal capillary pressure ranges from 16 mmHg to 32 mmHg (1 mmHg = 0.133 kPa). When persistent vertical pressure surpasses normal capillary pressure, capillary perfusion to the tissue is obstructed, resulting in tissue ischemia and hypoxia, which may eventually lead to ulceration or necrosis (22). Research has indicated that when an endotracheal tube is fixed in the oral cavity of patients, the head elevation and rotation can generate a maximum force of 3.5 N on the lips and face, corresponding to a pressure of approximately 260 mmHg/cm^2^ (34.58 kPa) on the oral soft tissues. External pressures exceeding 32 mmHg (4.27 kPa) can reduce blood flow, and those above 80 mmHg (10.67 kPa) can cause complete vascular occlusion (22, 41, 42). Previous finite element studies reported that a force of 3.43 N was applied to the lower lip by the endotracheal tube, resulting in maximum equivalent stresses of (41.72 + 1.14) kPa in the skin and mucosa and (50.12 + 1.01) kPa in the muscles.

In addition, studies have disclosed that increased shear forces at the interface between soft tissues and medical devices can exacerbate deformation caused by pressure alone (43–45). Internal stress and strain in tissues adjacent to bony prominence are significantly higher than those in tissues near the body surface. Furthermore, due to stress concentration effects, as the sharpness of the bony prominence increases, internal stress and strain also increase (46, 47). These stress concentrations may cause deep tissue injury before superficial tissue damage occurs or becomes clinically visible.

Beyond these biomechanical forces, several molecular and pathophysiological pathways contribute to mucosal injury. Sustained ischemia and reperfusion trigger excessive production of reactive oxygen species (ROS), leading to oxidative stress and activation of NF-κB–mediated inflammatory cascades (48). Upregulation of pro-inflammatory cytokines such as TNF-α, IL-1β, and IL-6 further amplifies tissue damage (49). Pressure- and shear-induced hypoxia also activate MAPK signaling (p38, ERK, JNK) (50), which promotes apoptosis through mitochondrial cytochrome c release and caspase-3 activation (51). Matrix metalloproteinases (e.g., MMP-2 and MMP-9) degrade extracellular matrix proteins and tight junction components, weakening epithelial barrier integrity and facilitating ulceration (52). Disruption of integrin- and cadherin-mediated adhesion further compromises cell–matrix interactions (53). During the repair phase, HIF-1α–driven VEGF expression stimulates angiogenesis (54), while TGF-β/Smad signaling regulates fibroblast activation and matrix remodeling (55). Together, these molecular mechanisms provide a pathophysiological link between mechanical loading and the initiation, amplification, and resolution of mucosal pressure injuries.

## 6 Injury prediction from a biomechanical perspective

FEA is a commonly used biomechanical modeling technique. By constructing three-dimensional finite element models of the oral mucosa based on computed tomography or magnetic resonance imaging data, it is possible to simulate stress distribution and injury processes under various conditions (56). FEA can also be applied to studies of oral mucosal injury by simulating stress distribution under different external forces to predict the likelihood of injury (57).

Risk prediction models based on biomechanical parameters and clinical data enable quantitative evaluation of the likelihood of oral mucosal injury (58). For example, one study used logistic regression analysis to identify independent risk factors for oral MMPI in patients undergoing orotracheal intubation, including intubation duration, catheter material, and frequency of daily oral care, and subsequently developed a predictive model. This model demonstrated high predictive accuracy and clinical applicability through validation with ROC and decision curve analyses (59). However, current applications remain largely theoretical, and further validation in clinical settings is required.

## 7 Application of biomaterial optimization in the prevention of mucosal injury associated with tracheal intubation

Biomaterial optimization refers to materials improved through bioengineering, chemical synthesis, or natural extraction to enhance their physical, chemical, and biological properties for superior performance in biomedical applications (60). In clinical settings, the application of low-friction coatings and prophylactic dressings can mitigate intubation-related oral mucosal injuries and reduce the risk of infection. A comparative summary of biomaterials and monitoring technologies, including their mechanisms, reported benefits, and limitations, is presented in Table 2 to provide clinicians with a concise overview.

**Table 2:** Comparative summary of biomaterials and monitoring technologies for preventing orotracheal intubation-related oral mucosal pressure injuries.

Category	Example(s)	Mechanism of action	Reported benefits/limitations
Hydrogel coatings	Silver-containing hydrogel; peptide-loaded hydrogels	Form lubricating layer; antimicrobial activity	Reduce friction and biofilm formation; limited long-term clinical validation
Nanocoatings	Silver nanoparticles; ZnO, Cu nanomaterials	Broad-spectrum antibacterial; anti-biofilm	Reduce VAP incidence; cost and safety require further evaluation
Foam dressings	Polyurethane foam (± silver)	Absorb exudate; reduce shear/friction	Maintain moist environment; frequency of replacement still debated
Hydrocolloid dressings	Pectin/CMC-based dressings	Occlusive, antimicrobial, promote debridement	Enhance wound healing; risk of maceration if overused
Monitoring technologies	Cuff-pressure monitoring; AI-based tube positioning	Real-time adjustment; predictive accuracy	Prevent mucosal ischemia, misplacement; need integration into ICU workflow

## 8 Low-friction coatings

### 8.1 Hydrogel coating

The hydrogel-coated endotracheal tube is a medical device derived from conventional endotracheal tubes with surface modification. Upon contact with water, it forms a lubricating layer resembling loach skin, which significantly reduces frictional resistance during intubation, minimizes damage to the airway mucosa, and enhances both the comfort and safety of the procedure. In addition, antimicrobial properties can be achieved by incorporating antimicrobial agents, such as silver ions or nanosilver, into the hydrogel coating. For instance, silver-containing double-network hydrogel coatings exhibit excellent antibacterial activity against common pathogens such as methicillin-resistant *Staphylococcus aureus* (MRSA) and *Pseudomonas aeruginosa*. Such an antimicrobial coating can effectively reduce bacterial colonization and biofilm formation on the catheter surface, thereby decreasing the incidence of ventilator-associated pneumonia (VAP) (61, 62). Early *in vitro* and animal studies suggest favorable biocompatibility, but clinical data remain scarce. Issues such as long-term durability, manufacturing complexity, and cost currently limit their widespread clinical use. In addition to silver-based hydrogel systems, other approaches include antimicrobial peptide-loaded hydrogels, photoreactive hydrogels, and multi-network hydrogels with combined lubricating and antibacterial properties, all of which have shown promise in reducing mucosal damage and bacterial colonization (62, 63).

### 8.2 Nanocoating

The nano-coated endotracheal tube is a novel medical device based on conventional endotracheal tubes, in which specialized nanomaterials are applied to the surface through nanotechnology (64). This approach primarily leverages the unique physical and chemical properties of nanomaterials, such as high specific surface area, antimicrobial activity, and biocompatibility, to enhance the performance of the endotracheal tube (65). Common nanocoating materials include silver nanoparticles, which exhibit broad-spectrum antibacterial activity, effectively inhibit bacterial growth, and prevent biofilm formation; nanocomposites, such as polyacrylamide-gelatin coatings, which provide both antibacterial and antifouling properties; and other nanomaterials, such as silica nanoparticles, which improve coating stability and biocompatibility (66). Research has demonstrated that nanocoatings can effectively inhibit bacterial colonization on the surface of endotracheal tubes, thereby reducing the incidence of infections such as VAP. These coatings can prevent the adhesion of proteins, cells, and platelets, lowering the risk of lumen obstruction. In addition, certain nanocoatings exhibit lubricant properties, which help reduce friction and tissue damage during intubation (67, 68). Silver and other nanoparticle-based coatings have shown efficacy in reducing bacterial colonization and biofilm formation in both experimental and limited clinical trials (e.g., the NASCENT trial). However, concerns remain regarding potential cytotoxicity, resistance development, and higher production costs. Beyond silver nanoparticle coatings, various other nanomaterials such as zinc oxide, copper, and silica nanoparticles, as well as polymer-based nanocomposites, have been investigated. These materials not only provide antibacterial and antibiofilm functions but also improve coating stability and biocompatibility, further broadening the scope of clinical applications for coated endotracheal tubes (64, 66–68).

## 9 Prophylactic dressings

### 9.1 Foam dressings

Foam dressings are primarily composed of polyurethane foam, with some products incorporating antibacterial agents such as silver ions. Their key properties include excellent fluid absorbency and breathability, enabling effective exudate absorption while maintaining a moist wound environment. Further, the addition of bioactive components, such as growth factors, promotes wound healing (69–74). In addition, foam dressings feature a soft texture that helps reduce frictional trauma to the wound. They are designed with a multilayer structure, where the outer layer absorbs exudate upon direct wound contact, the intermediate layer facilitates breathability, and certain silver ion-containing products provide antimicrobial properties, reducing the risk of infection (75). In clinical practice, plasma treatment can be applied to the surface of foam dressings to enhance surface activity, thereby improving their adherence to wound tissue and increasing fluid absorption efficiency. Additionally, grafting bioactive molecules such as antimicrobial peptides onto the foam dressing surface enhances antibacterial properties, potentially reducing the need for antibiotic use. Personalized foam dressings can be designed based on wound morphology and size to improve adherence, decrease material waste, and reduce the frequency of dressing changes, thereby mitigating the risk of infection. Foam dressings are widely applied clinically and are generally safe, but frequent replacement is required and costs may accumulate, particularly in ICU patients requiring prolonged intubation. Clinical trials should be conducted to optimize the application techniques and replacement frequency of foam dressings, minimizing secondary damage to the wound (76).

### 9.2 Hydrocolloid dressings

Hydrocolloid dressings are typically composed of hydrophilic polymers such as pectin and sodium carboxymethyl cellulose, providing excellent occlusive and antimicrobial properties, which help reduce the risk of wound infection (77). Upon contact with wound exudate, these dressings form a gel, maintaining a moist wound environment, promoting autolytic debridement, and facilitating the breakdown and detachment of necrotic tissue. The excellent adhesion of hydrocolloid dressings to the wound and surrounding skin reduces mechanical friction and external trauma, while also offering some antibacterial properties to lower the incidence of infection (78). In clinical applications, nanotechnology is employed to construct nanostructures on the surface of hydrocolloid dressings, increasing their specific surface area to enhance exudate absorption and drug-loading capacity. Surface modification with anti-inflammatory factors further endows these dressings with anti-inflammatory properties, accelerating wound healing (79). Hydrocolloids promote moist wound healing and are safe in most settings; however, the risk of local maceration and the need for regular monitoring represent practical limitations.

## 10 Impact of oral microecosystem on tracheal intubation-associated airway injury

### 10.1 Tracheal intubation and oral microecosystem

Tracheal intubation is a common clinical procedure used to maintain airway patency; however, it may also affect the oral microecosystem, thereby increasing the risk of respiratory tract infections. The oral cavity represents a complex and complete microbial ecosystem that supports the survival of diverse microorganisms. Disruption of this microecological balance creates favorable conditions for the colonization and proliferation of various microorganisms, including bacteria, fungi, mycoplasma, viruses, and protozoa (75). The oral cavity is a major entry point for bacteria that invade the respiratory tract and lungs, where pathogenic bacteria first colonize mucosal sites, including the oropharynx and nasopharynx. Following sustained adhesion and proliferation, these pathogens reach the lower respiratory tract and lungs via aspiration or inhalation, ultimately triggering corresponding pathological responses (76). Research indicates that numerous microorganisms coexist in the oral cavity, where they maintain ecological balance through mutual restriction and interdependence. Tracheal intubation, as an invasive procedure, disrupts the normal distribution of oral microbiome, leading to dysbiosis (77). For example, certain beneficial bacteria may be suppressed, while harmful bacteria may proliferate excessively. In addition, the endotracheal tube surface and its surrounding environment provide a favorable niche for bacterial growth. Studies have shown that bacteria can adhere to the surface of endotracheal tubes in the form of biofilms, which protect them from both immune defenses and antibiotic treatment, leading to extensive colonization in the oral cavity. Consequently, these bacteria can more easily migrate through the tube surface and airway gaps into the lower respiratory tract and even the lungs, causing respiratory infections such as VAP (78, 79).

### 10.2 Effect of microorganisms on oral mucosal injury and repair

The oral microbiome, in coordination with the mucosal immune system, epithelial barrier, and other factors, establishes a complex, diverse, and dynamic balance referred to as oral mucosal homeostasis. Under homeostatic conditions, the oral mucosal immune response selectively recognizes and facilitates the symbiotic colonization of microorganisms, while in turn, the oral microbiome promotes the development and maturation of oral immune cells. Epithelial barrier cells express various pattern recognition receptors that participate in microbial responses; and the oral microbiome dynamically influences the structure and function of host epithelial cells (80). Conversely, oral dysbiosis refers to a process in which environmental disturbances disrupt microbial homeostasis beyond the host's tolerance and self-repairing capability, ultimately leading to functional alterations of the microbial ecosystem. In recent years, increasing evidence has shown that microbial dysbiosis contributes to the occurrence and progression of oral mucosal diseases. For example, *Porphyromonas gingivalis*, a pathogenic bacterium, produces gingipains that induce host matrix metalloproteinases to enhance collagen degradation, affecting N-cadherin, VE-cadherin, and β-integrin, thereby reducing cell adhesion to extracellular matrix proteins. This process leads to epithelial cell detachment and ultimately compromises the integrity of the epithelial barrier (81–83). In addition, *Candida albicans* can induce calcium influx and lactate dehydrogenase release in epithelial barriers, leading to epithelial cell damage. Candidal hemolysin secreted by *Candida albicans* damages mucosal epithelial cells and activates mitogen-activated protein kinase signaling mediated by the epidermal growth factor receptor family, leading to neutrophil recruitment and the production of inflammatory cytokines (84). Recent progress in microbiome sequencing has revealed that microbial dysbiosis is also involved in oral mucosal repair. Under normal conditions, abundant commensal microorganisms in the oral cavity suppress pathogen overgrowth by competing for nutrients and habitat, maintaining microecological balance and supporting mucosal tissue repair. Certain beneficial microorganisms can promote mucosal epithelial cell proliferation and differentiation by modulating the host immune response, thereby accelerating the healing of damaged sites. Furthermore, the oral microbiota plays a critical role in mucosal repair by regulating mucosal immune responses. For instance, certain microorganisms can stimulate immune cells to release cytokines, regulating both the intensity and duration of the inflammatory response, thereby preventing excessive inflammation-induced tissue damage while facilitating immune cell migration to the injury site to enhance tissue repair (85–87).

Nevertheless, it should be noted that the present discussion has mainly focused on dysbiosis literature from oral lichen planus and recurrent aphthous stomatitis. These examples may not fully represent the broader microbial alterations associated with orotracheal intubation and ICU-related conditions. Dysbiosis has also been implicated in ventilator-associated pneumonia, peri-implant mucositis, and other forms of mucosal barrier dysfunction, but direct evidence linking these findings to oral MMPI remains limited. Thus, the scope of this review is restricted, and future studies should integrate microbiome changes across a wider range of clinical contexts to provide a more comprehensive understanding of their role in mucosal injury and repair.

## 11 Comprehensive intervention strategies based on multidimensional integration

### 11.1 Biomechanical optimization

Endotracheal intubation is an essential intervention for mechanical ventilation in ICU patients; however, the endotracheal tube itself and improper fixation methods can lead to the development of oral MMPI (21). From a biomechanical perspective, the primary forces contributing to oral MMPI caused by endotracheal tubes include vertical pressure, shear forces, and friction. Continuous vertical pressure exerted directly on the mucosal tissues is the main cause of pressure injuries, and the occurrence of oral MMPI is associated with both the magnitude and duration of pressure. Higher pressure levels and prolonged compression increase the risk of oral MMPI (22). Research indicates that shear force is defined as a force applied to the surfaces of adjacent tissues, causing them to slide in opposite directions. The shear force results from the combination of pressure and friction and is generated when the endotracheal tube contacts the oral soft tissues. Once shear force acts on the deep layers of the skin, it can stretch, twist, and rupture capillaries, interrupting local blood flow and causing circulatory disturbances, eventually leading to deep tissue necrosis. Such injuries are difficult to identify in the early stages and are more difficult to heal than typical wounds. Friction arises from the relative displacement between the surfaces of the oral mucosa and the endotracheal tube. Although friction itself does not directly induce oral MMPI, it can damage the stratum corneum, causing superficial mucosal exfoliation and enhancing sensitivity to pressure-related injuries. The exposed mucosa, when irritated by saliva and other secretions, faces an increased risk of pressure injuries. Moreover, friction elevates local mucosal temperature, disrupts microenvironmental homeostasis, alters pH levels, and increases oxygen consumption, thereby intensifying ischemia and further raising the risk of oral MMPI [81]. Clinically, endotracheal tubes should be fixed at the corner of the mouth to maximize the contact area between the tube and the oral commissure, while positioning the tube in the central part of the oral cavity to minimize both the contact area and duration of contact between the tube and the oral mucosa. Additionally, regular repositioning of the fixation site is recommended to redistribute pressure, and measures should be taken to alleviate patient agitation, thereby reducing pressure and shear forces on the oral mucosa, which can help mitigate mucosal injury associated with endotracheal intubation to some extent (22).

### 11.2 Oral microecosystem regulation

Mucosal injury caused by endotracheal intubation is closely associated with oral microecological imbalance. The oral microecosystem refers to the dynamic equilibrium formed by interactions between oral microbial communities and the host, which plays a critical role in maintaining oral mucosal health. Disruption of this balance can lead to mucosal injury, whereas regulating the oral microecosystem can effectively alleviate mucosal injury (88). Research has demonstrated that some probiotics, such as *Lactobacillus* species, can produce antimicrobial or protective substances within the oral cavity, including organic acids, hydrogen peroxide, bacteriocins, short-chain fatty acids, antimicrobial peptides, and reuterin (89). These antimicrobial substances can inhibit the growth of pathogenic bacteria in the oral cavity and reduce their toxicity to the host. Bacteriocins can inhibit the formation of biofilms by pathogenic bacteria and reduce the levels of pro-inflammatory factors such as cytokines, collagenase, elastase, and prostaglandin E2. This promotes stronger adhesion of probiotics to the oral biofilm surface and prevents colonization by new pathogens, thereby establishing a new microbial balance and forming a healthy biofilm (90). Short-chain fatty acids can alter the transmembrane pH gradient, thereby inhibiting pathogen growth, while antimicrobial peptides contribute to bacterial lysis through insertion into pathogen cell membranes (91). Reuterin produced by *Lactobacillus reuteri* helps maintain oral microbial homeostasis by neutralizing lipopolysaccharides from *Porphyromonas gingivalis*, thereby supporting mesenchymal stem cell migration, self-renewal, osteogenesis, and proliferation, while inhibiting inflammation and promoting wound healing (92).

### 11.3 Intelligent monitoring and personalized care

With the development of technologies such as artificial intelligence and the Internet of Things, intelligent monitoring and personalized care for endotracheal intubation have gradually become research hotspots in the medical field. Relevant technologies include airway pressure and ventilation parameter monitoring, cuff-pressure monitoring, intubation position monitoring, and remote monitoring with early warning systems (93–96). Intelligent monitoring systems are capable of real-time monitoring of parameters such as airway pressure, positive end-expiratory pressure, tidal volume, and the ventilation–perfusion ratio. Through machine learning–based data analysis, these systems provide early warnings for healthcare providers, optimize ventilation strategies, and reduce the incidence of ventilator-associated lung injury and other complications. For example, closed-loop control of mechanical ventilation, such as Evita 4 and Galileo, can automatically adjust ventilator settings based on real-time physiological data from the patient, providing personalized ventilation support. Intelligent cuff-pressure monitoring systems can automatically regulate cuff-pressure to maintain it within a safe range, thereby reducing the risk of tracheal mucosal injury and aspiration caused by excessive or insufficient cuff-pressure. Some systems are also equipped with subglottic secretion drainage functions, which monitor the accumulation of secretions above the cuff and automatically perform irrigation and suction to reduce the risk of infection. Intubation position monitoring utilizes predictive models based on artificial intelligence to analyze multiple clinical parameters and provide precise recommendations for intubation depth, thereby reducing the risk of misplacement. Certain intelligent devices employ image recognition technology to monitor tube position in real time, ensuring accurate tracheal placement and providing visual guidance during the procedure. Remote monitoring and early warning systems based on the Internet of Things can transmit real-time patient data to healthcare providers' terminals, enabling remote surveillance and timely intervention. These systems also feature automatic alarms for conditions such as abnormal cuff-pressure and accidental extubation, promptly notifying healthcare personnel to take corrective actions. In addition, intelligent nursing systems can formulate personalized care plans based on patient data, including health status, medical history, and real-time monitoring parameters, with dynamic adjustments according to changes in the patient's condition. For example, targeted nursing interventions can be designed to address the respiratory management, airway clearance, and psychological needs of patients with endotracheal intubation (97, 98).

Comprehensive intervention strategies should therefore integrate multiple dimensions, including biomechanical optimization (e.g., adjusting tube positioning and fixation to redistribute pressure), oral microecosystem regulation (e.g., using probiotics to restore microbial balance), and intelligent monitoring systems (e.g., cuff pressure monitoring and personalized care plans). Proposed AI/IoT systems for orotracheal-intubation care can leverage well-established supervised algorithms for event prediction (e.g., random forest, gradient boosting machines, and regularized logistic regression) and deep models for signal and image streams (e.g., one-dimensional convolutional neural networks and long short-term memory networks for time-series airflow/cuff-pressure data; lightweight convolutional neural networks for tube-position images) (99, 100). Feature sets typically include cuff-pressure traces, ventilator loops, motion indices, tube-position signals, and contextual clinical variables. Data integration may follow early-fusion, late-fusion, or intermediate-fusion strategies (101), while Kalman or Bayesian filtering can smooth noisy signals (102).

Clinical validation should report discrimination (AUROC, AUPRC), sensitivity, specificity, calibration, and decision-curve net benefit (103, 104). Internal validation may rely on stratified cross-validation, whereas external validation requires temporally or geographically distinct ICU cohorts. Prospective pilots, such as those used in AI sepsis management (105), could also quantify real-world impact.

## 12 Future outlook

As artificial intelligence and Internet of Things technologies progress, intelligent monitoring devices will become increasingly precise, convenient, and efficient. Improvements in AI algorithms will enhance the accuracy of airway evaluation and monitoring, and innovations such as wearable monitoring devices and remotely operated intubation robots can broaden the application scenarios of endotracheal intubation. Furthermore, intelligent monitoring and personalized nursing care for endotracheal intubation require close collaboration across multiple disciplines, including anesthesiology, critical care, nursing, and artificial intelligence, to develop more comprehensive and effective solutions through interdisciplinary research.

In addition, although certain microbial metabolites have been identified to regulate the mucosal barrier, the underlying molecular mechanisms remain incompletely understood. Future research should further investigate the signaling pathways between the microbiota and host cells, including how microbial metabolites interact with host cell surface receptors and how these signals are transmitted intracellularly to regulate gene expression and cellular functions. Simultaneously, novel functional bacterial strains and metabolites should be identified through metagenomics and metabolomics approaches to discover more strains and metabolites with mucosal barrier repair capabilities and explore their mechanisms of action under different disease conditions. However, due to interindividual differences in microbiota composition and function, it is necessary to develop precision microecological interventions tailored to individual microbial profiles. By analyzing the composition and function of the microbiota in each individual, personalized probiotic formulations or dietary interventions can be designed to more effectively restore the mucosal barrier.
